# Anesthetic Management of the Surgical Correction of Idiopathic Scoliosis in a Teenager With Ornithine Transcarbamalyse Deficiency

**DOI:** 10.7759/cureus.45393

**Published:** 2023-09-17

**Authors:** Sérgio G Pinto, Patrícia Martins Lima, José Dias

**Affiliations:** 1 Anesthesiology, Centro Hospitalar Universitário São João, Porto, PRT

**Keywords:** pediatric orthopedic surgery, caloric supplementation, urea cycle disorder, idiopathic scoliosis, locoregional anesthesia, combined anesthesia, metabolic disorder, propofol infusion, erector spinae plane block, otc deficiency

## Abstract

Ornithine transcarbamylase (OTC) deficiency is the most common genetic disorder of the urea cycle. These disorders are characterized by an inability to metabolize ammonia into urea, leading to hyperammonemia with variable physiological consequences and presenting important anesthetic challenges, especially the perioperative prevention of hyperammonemia and management of its consequences, should it occur. Idiopathic scoliosis (IS) is the most common spinal deformity requiring surgical treatment. This paper presents the case of a 16-year-old female with OTC deficiency who underwent spinal fusion for IS. The chosen anesthetic strategy was combined anesthesia with total intravenous general anesthesia using target-controlled infusion pumps, an erector spinae plane block (ESPB), and a multi-pronged approach to ensure metabolic control while avoiding hyperammonemia. The existing literature regarding major surgery in patients with OTC deficiency is sparse, and this paper provides one of the first case reports of a scoliosis correction surgery, as well as one of the first descriptions of prolonged propofol infusion and locoregional anesthesia with an erector spinae plane block in this context.

## Introduction

Ornithine transcarbamylase (OTC) deficiency is a genetic disorder with an X-linked pattern of inheritance that is defined by a partial or complete deficit of OTC, which is an essential enzyme in the urea cycle [1]. It is the most frequent among the urea cycle disorders, which are characterized by an inability to metabolize ammonia into urea, leading to hyperammonemia with a wide range of both short- and long-term physiological consequences [2]. Ornithine transcarbamylase deficiency is a highly heterogeneous disease with great variability in both clinical manifestations and age of onset [3]. Severe deficiencies are more common in males and typically present in the neonatal period, with serious consequences for hyperammonemia and a risk of permanent neurological damage. Partial deficiencies have the greatest possible clinical spectrum and are common in heterozygous females [4]. In female carriers, severe symptoms in childhood are rare; up to 20% can experience mild symptoms, and some individuals do not experience any symptoms until a stressor, such as pregnancy or delivery, triggers an acute episode of hyperammonemia [3]. Among the common symptoms of OTC deficiency are gastrointestinal and neurological manifestations. Hyperammonemic episodes present as vomiting, lethargy, irritability, confusion, delirium, and ataxia, with a risk of life-threatening complications if untreated [3,5].

Idiopathic scoliosis (IS) is a spinal deformity that is defined by a lateral curvature (the Cobb angle) of 10º or more in relation to its central axis and frequently accompanied by complex three-dimensional changes to the spinal anatomy, with no known cause [6]. It is the most common of spinal deformities, and has a highly protean age of onset and clinical presentation, ranging from indolent and benign to rapidly progressive and potentially fatal. Surgical correction is often performed via spinal fusion, which is a complex procedure associated with an important risk of perioperative complications as well as important intraoperative blood loss and difficult postoperative pain control [7,8].

## Case presentation

The patient described in this case is a 16-year-old female with OTC deficiency and IS. At approximately five years of age, the patient was admitted to the hospital after a six-month period of post-prandial vomiting, anorexia, and weight loss. Additionally, the transaminases were elevated, and the patient’s blood ammonia was 505 ug/L. The patient had a compatible family history of OTC deficiency, and further testing confirmed the diagnosis. Currently, the patient’s illness is well controlled with a therapeutic regimen (Table 1) and a restrictive diet.

**Table 1:** The patient’s therapeutic regimen

Drug	Dose	Posology
Sodium benzylbutyrate	3 g	Three times a day
Sodium phenylbutyrate	2.5 g	Three times a day
Arginine	3 g	Once daily
Folic acid	5 mg	Once every other week
Oral iron	100 mg	Once every other day
Valine, isoleucine, and leucine	100 mg	Once daily
Excitatory amino acids	10 g	Once daily

**Figure 1:**
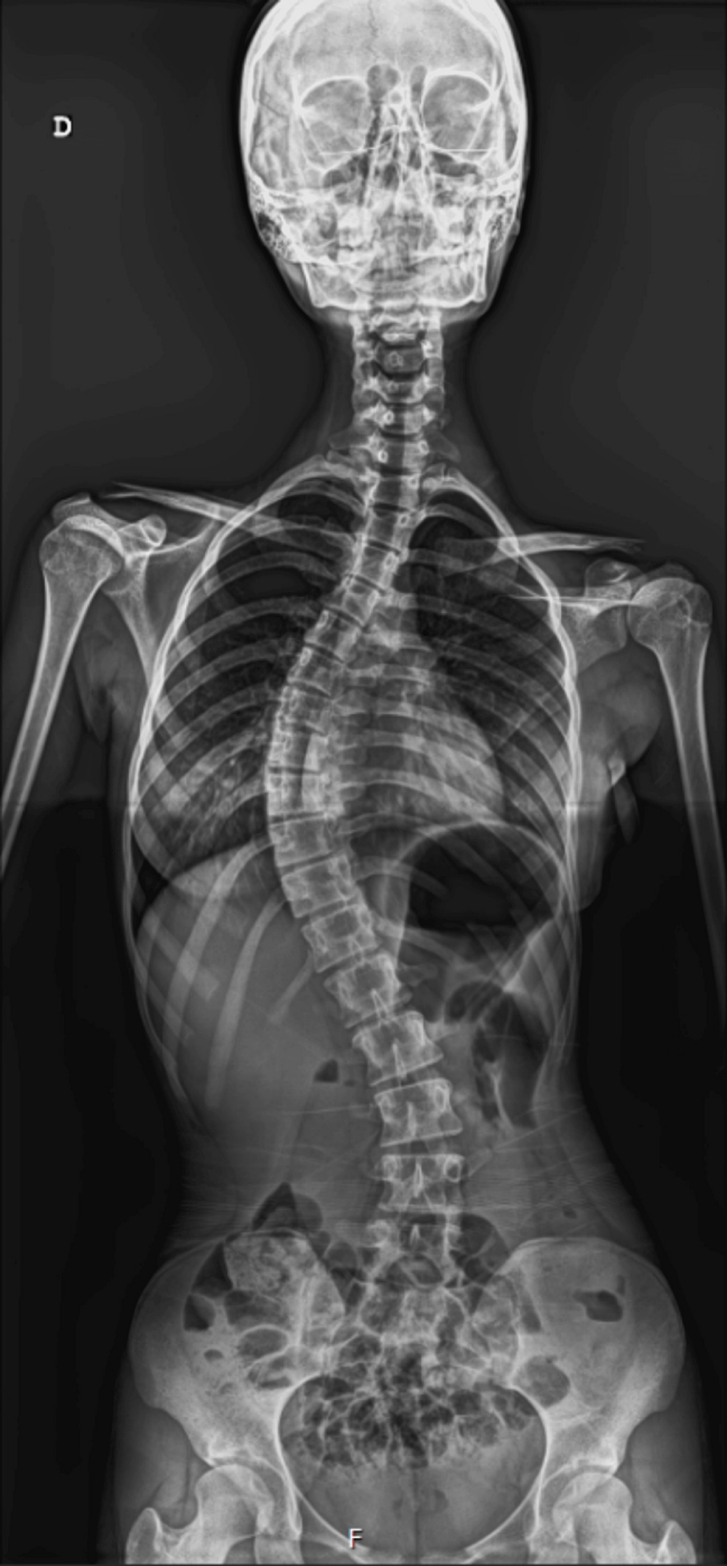
The patient's preoperative X-ray

At the age of eight, the patient was diagnosed with IS with a thoracolumbar extension spanning 12 vertebral levels and a Cobb angle of 16º. She was kept under clinical surveillance from ages eight to 12. From ages 12 to 16, she was treated with a Boston vest to prevent progression. At the age of 16, with a Cobb angle of 38º (Figure 1), the patient was enrolled on the surgical waiting list for a spinal fusion in a regional tertiary hospital.

The patient weighed 38.5 kg with a height of 151 cm, placing her in the fifth to 15th percentile body mass index range for her age.

As part of her surgical preparation regimen, the patient was admitted to the hospital the day before the surgery and underwent a standard surgical fast while under infusions of sodium benzoate (10 g diluted in 430 mL of 10% glucose) at a rate of 20 mL/h and of 10% glucose at a rate of 50 mL/h, which were maintained up to and throughout the surgery. A total intravenous general anesthesia (TIVA) was performed using target-controlled infusion (TCI)-ready infusion pumps, using the Paedfusor-effect model for the propofol infusion and the Minto-effect model for the remifentanil infusion. Anesthetic induction was performed via the TCI-ready infusion pumps with a target concentration of 6 µg/mL for propofol and 3 ng/mL for remifentanil and with the manual administration of 20 mg of rocuronium. The patient was monitored according to the American Society of Anesthesiologists Standards for Basic Anesthetic Monitoring, as well as anesthetic depth monitoring with a bispectral index (BIS) monitor and neuromuscular blockade monitoring with a kinemyiography train-of-four device. The surgical technique required the monitoring of motor and sensitive evoked potentials.

The airway was secured with an armored orotracheal tube placed via direct laryngoscopy, an arterial line was placed in the left radial artery for invasive blood pressure (BP) monitoring, and a triple lumen central venous catheter was placed in the patient’s right internal jugular vein via an ultrasound-guided Seldinger technique.

The patient was rotated to ventral decubitus. Gel cushions were used to position the patient while ensuring that the abdomen, axillae, eyes, and nose remained free of pressure and that sensitive pressure points, such as the bony prominences of the hip, knees, ankles, and elbows, were adequately protected and cushioned.

Before the incision, an infusion of sodium chloride (NaCl) 0.9% at a rate of 250 mL/h was initiated, 800 mg of paracetamol was administered, and an ultrasound-guided erector spinae plane block (ESPB) with a bilateral injection of 20 mL of 0.2% ropivacaine (totaling 80 mg) was performed at level T9. All infusions were placed within fluid warming systems.

The surgery duration was 135 minutes. The patient was hemodynamically stable throughout the surgery, with an average mean arterial pressure of 73 mmHg and an average heart rate of 68 bpm. Anesthetic depth remained within the target range of 40-60 BIS. Normothermia was assured, with an average temperature of 36ºC. The mean blood glucose was 128 mg/dl. Blood was drawn for an ammonia test 90 minutes into the surgery, with a result of 35 µmol/L, which was inferior to the 44 µmol/L recorded in the preoperative study.

During the surgical procedure, 1500 mg of propofol, 2 mg of remifentanil, and 30 mg of rocuronium were administered. At the end of the surgery, 4 mg of morphine and 4 mg of ondansetron were administered, and the patient was rotated back to dorsal decubitus. The patient suffered a total fluid loss of 625 mL, divided between urinary losses of 310 mL and hemorrhagic losses of 315 mL, for a total positive fluid balance of 95.45 mL. She left the operating room (OR) intubated under mechanical ventilation with infusions of propofol and remifentanil and was transported to the pediatric intensive care unit (PICU), where she was extubated with no complications two hours after the surgery.

**Figure 2:**
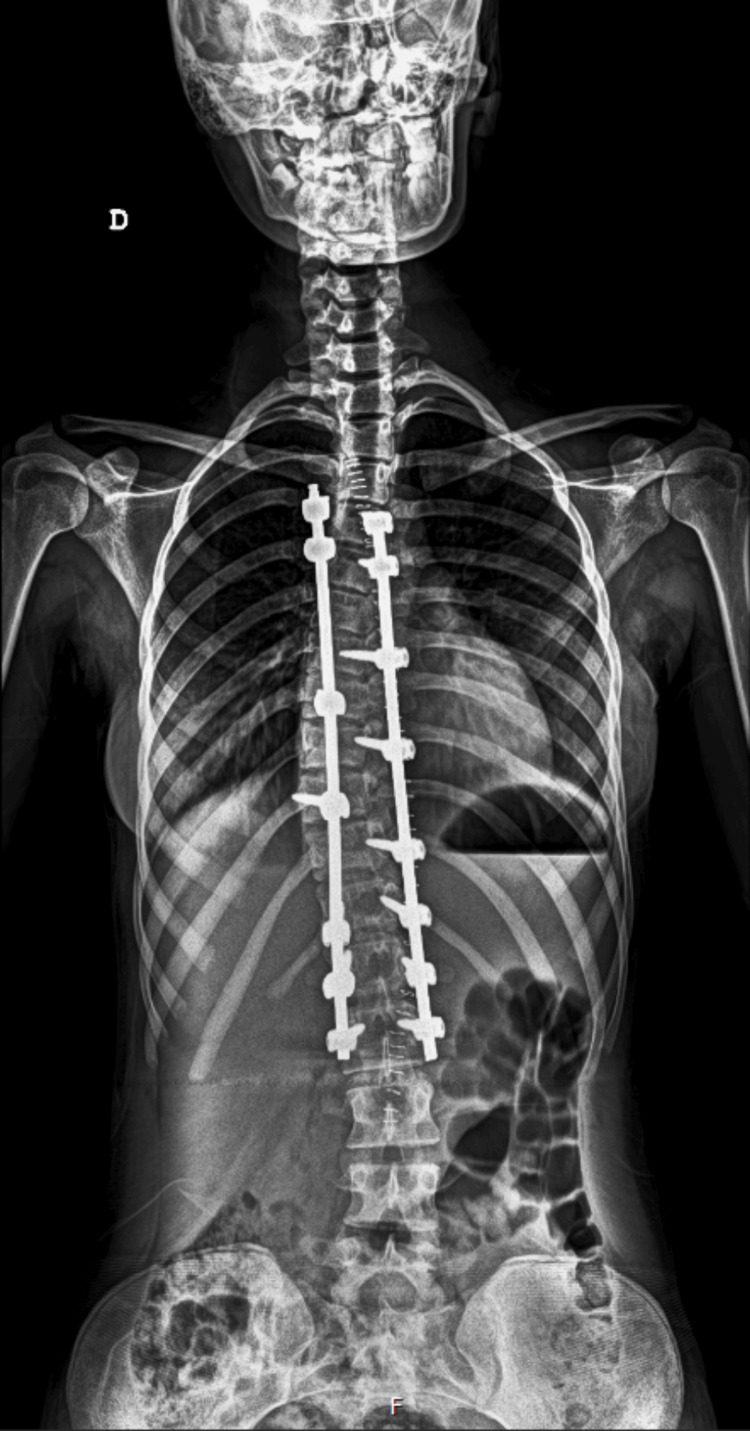
The patient's X-ray at discharge

The patient was instructed in the usage of a morphine patient-controlled anesthesia (PCA) device (1 mg/mL; 1 mL bolus; 8-min lockout). Throughout her stay in the PICU, the patient opted to use approximately 61% of the total possible PCA boluses and no rescue analgesia. Pain was measured using the visual analogue scale with a measurement of 4/10 at rest and 6/10 in movement at six hours and 3/10 at rest and 5/10 in movement at 12 hours after the surgery. The patient was on bed rest for the first three days after the surgery and was discharged six days postoperatively with no complications. Imaging studies before discharge demonstrated a significant improvement in the patient’s spinal deformity, as shown in Figure 2.

Six months after the surgery, the patient was satisfied with the results of her treatment, with both functional and psychological improvements. There was no negative impact on the metabolic control of the patient’s OTC deficiency, which remains well controlled with the same medication regimen as detailed before.

## Discussion

This case report represents the first case of scoliosis correction surgery in a patient with an OTC deficiency. As scoliosis is not comorbid with OTC deficiency, this was a rare coincidence of two unrelated pathologies superimposed upon each other.

The patient’s OTC deficiency mandates important considerations that would not otherwise be present in a standard case of spinal fusion for IS. The cornerstone of anesthetic management in these patients is metabolic control, prevention of protein catabolism, and prevention of hyperammonemia [9], demanding a multidisciplinary perioperative strategy as discussed below.

The patient is under the care of a pediatrician who specializes in metabolic diseases and is on a strict diet and medication regimen, as detailed in Table 1. This, coupled with regular laboratory control of liver function, blood ammonia, and electrolyte levels, ensured that the patient was metabolically stable prior to surgery.

In addition to her regular diet and medication regimen, the immediate preoperative period was also discussed with the patient’s metabolic pediatrician, and the decision was made for the patient to undergo a standard surgical fast while under infusions of 10% glucose and sodium benzoate to protect against protein catabolism. Sodium benzoate is an essential drug in the treatment of OTC deficiency and other urea cycle disorders by providing an alternate route for the elimination of nitrogenous residues, as it is conjugated with glycine to form hippurate, thus allowing for the renal excretion of the trapped ammonium ions [10]. These infusions were maintained throughout the surgery for the same effect.

Sevoflurane is the most common choice as a maintenance agent for patients with OTC deficiency undergoing surgery, as described in the review published by Del Rio et al. [9]. However, spinal fusion for IS requires monitoring of motor and sensitive evoked potentials [7], and evidence shows that halogenated gases, like sevoflurane and desflurane, have a dose-dependent inhibitory effect on the aforementioned motor and sensitive evoked potentials [11], making them undesirable in this context.

While the use of propofol as an agent for the maintenance of anesthesia has been described in a patient with carbamoyl phosphate synthetase I deficiency who underwent anesthesia for a short duration, this was the first case report describing a prolonged TIVA-TCI in a patient with OTC deficiency [9].

Prolonged propofol infusions are contraindicated in many metabolic disorders (e.g., organic acidemias). However, in disorders of the urea cycle (e.g., OTC deficiency), no physiological basis exists to contraindicate their use. On the contrary, propofol offers several apparent benefits in this context, namely its protective effect on whole-body protein catabolism [12] and as an additional source of caloric supplementation thanks to the lipid solution it is prepared in [13].

Caloric supplementation during surgery is an accepted and exceptionally important practice to protect patients with urea cycle disorders from the increased protein catabolism inherent to the surgical state. Lipid infusions (with intralipid) and glucose infusions are described; their caloric values are tabled as 1.1 kcal/mL for intralipid 10%, 2 kcal/mL for intralipid 20%, and 0.4 kcal/mL for glucose 10%. In comparison, propofol has a caloric value of 1.1 kcal/mL, which is equal to a low-concentration lipid infusion. In this case, the infusion of propofol contributed a total of 165 kcal to the patient in comparison to the 112.5 kcal supplemented by the glucose infusion, highlighting the added utility of propofol infusions in this context as an additional line of defense against protein catabolism [12,13].

The anatomical distortion that is characteristic of IS poses a challenge to the execution of the ESPB in this context. However, significant evidence exists that single-shot ESPB is an efficient and safe technique for use as part of a multimodal analgesia regimen in spinal fusion surgery [14]. Adding to its favorable characteristics, this technique does not appear to interfere with motor and sensitive evoked potential monitoring [15].

The existing literature is also sparse in its descriptions of locoregional anesthesia with ropivacaine in this context [16]. Apart from its beneficial pharmacological and selectivity profile, ropivacaine metabolism occurs via CYP1A2 in the liver and has no interaction with the urea cycle [17], and as such, there is no physiologic basis contraindicating its use in these patients. We used ropivacaine for the execution of the erector spinae plane block in this patient with no negative impact on ammonia control.

Antiemetic prophylaxis for surgery is not a consensual issue in the context of OTC deficiency, with some authors claiming that the benefit of vomiting as an early sign of hyperammonemia outweighs the benefit of antiemesis [18]. In this case, in addition to the protective effect granted by TIVA, we opted to use ondansetron as an antiemetic prophylaxis, as it is safe and has proven efficiency in OTC deficiency. In our analysis, the potential negative impact of postoperative nausea and vomiting on the patient’s recovery far outweighs any possible benefits. To screen for hyperammonemia, tight control of blood ammonia levels was obtained with laboratory analysis and close clinical surveillance in the PICU.

Intraoperative hemorrhagic control was satisfactory; however, a postoperative drop in blood hemoglobin demanded the transfusion of 1 unit of erythrocyte concentrate. While it is standard practice to use tranexamic acid in spinal fusion surgeries in our hospital, we decided against its use in this case as it has a known association with hyperammonemia [9,19]. Aminocaproic acid is not sufficiently studied in the context of urea cycle disorders, and as such, we decided not to use it. However, it would be of great interest to explore this drug’s safety in the context of urea cycle disorders as a possible means of hemorrhagic prevention, not only in major surgery but also in trauma and other contexts, given the known risks of erythrocyte transfusions.

In the postoperative period, the patient’s pain was effectively controlled with moderate usage of her morphine PCA and without the need for rescue analgesia.

This rarity of this patient’s situation presents a series of important ethical considerations, owing to the natural uncertainty that surrounds rare cases with scarce descriptions in the literature. Throughout the course of her medical follow-up, the patient and her legal guardian were included in the discussions of metabolic, surgical, and anesthetic strategies and the benefits, risks, and uncertainties thereof. Informed consent was obtained at every step.

It is essential to note that this paper represents a single case report with all inherent limitations, namely, difficulty in generalizing our findings to the wider population of patients with OTC deficiency. While our experience provides evidence in favor of the safety and efficiency of these techniques, namely long propofol infusions and locoregional anesthesia using ropivacaine, for use in the context of spinal fusion surgery in patients with OTC deficiency, the authors are of the opinion that further research is paramount in identifying the ideal strategies for these patients.

## Conclusions

Major surgery in patients with OTC deficiencies presents a significant challenge to the anesthesiologist. The metabolic management of these patients, centered around the prevention of hyperammonemia, requires an attentive multidisciplinary perioperative approach.

This case report highlights the safety and benefits of TIVA-TCI techniques that rely on long propofol infusions for surgery in patients with OTC deficiencies, as well as the successful and safe usage of ESPB with ropivacaine for pain control, while also demonstrating an efficient perioperative metabolic protection regimen that successfully prevented hyperammonemia. 
